# COVID-19 postmortem neuropathology: insights of a meta-analysis of 464 cases

**DOI:** 10.1007/s10072-025-08320-6

**Published:** 2025-06-25

**Authors:** David S. Younger

**Affiliations:** 1 Department of Medicine, Section of Internal Medicine and Neurology, White Plains Hospital, White Plains, NY USA; 2 Department of Clinical Medicine and Neuroscience, CUNY School of Medicine, New York, NY USA

Dear Editor,

This meta-analysis concerns 464 decedents of 2019 coronavirus disease (COVID-19) who underwent detailed postmortem pathological studies due to severe acute respiratory syndrome-coronavirus-2 (SARS-CoV-2) as reported in four serial literature case reviews [1–4] by a single author.

The initial cohort of 50 postmortem literature cases reviewed by Younger [1] comprised older age subjects (age > 65 years in 79%, with men outnumbering women (3:1) and no pediatric cases. Elevated procoagulant and D-dimer levels increased the risk of brain infarction, as noted in 6 cases (12%), classified as hemorrhagic (2 cases) or ischemic (4 cases). Immuno-histochemical (IHC) localization of SARS-CoV-2 in the olfactory bulbs employing in situ polymerase chain reaction (PCR) of fresh frozen paraffin embedded tissue (PEFFT) blocks identified the nasal mucosal interface as the likeliest mode of CNS invasion. Interstitial lymphocytic inflammation was noted in the brainstem of 6 cases (12%) and leptomeninges in 7 cases (14%), in conjunction with terminal hypoxia–ischemia so noted in only one-half of cases overall.

A subsequent literature review of decedents by Younger [2] in early 2022 added 91 published decedents (totaling 141 cases) that showed three other salient findings. First, positive SARS-CoV-2 RNA genomes in 24 of 85 cases (28%) studied by quantitative real-time (qRT)-PCR suggested active viral replication outside the respiratory tract. Second, interstitial cytotoxic T-cells in 41 cases (45%) was detected by flow cytometry IHC, notably in leptomeningeal and brainstem tissue sections, with rare examples of microglial activation as indicated by positive staining for the human leukocyte antigen-DR isotype. Third, there was increased expression of angiotensin converting enzyme 2 (ACE2) in oligodendrocytes, and transmembrane serine proteases 2 and 4 (TMPRSS2 and TMPRSS4) expression in neurons, respectively coding proteins implicated in viral entry (ACE2) and pruning of the viral-decorating spikes (TMPRSS2).

By the end of 2022, Younger [3] summarized 150 additional decedents, adding 12 children and 138 adults of age range 7 months to 97 years, with three other notable findings. First, childhood fatalities included 7 cases of multisystem inflammatory syndrome (MIS-C), and another with angiographically-negative, small vessel childhood primary angiitis of the CNS (SVcPACNS). Second, the occurrence of acute hemorrhagic and thrombotic infarcts was increasing (11% and 25%, versus 2% and 7%), compared to the earlier cohort [2], in association with a modest increase in interstitial brainstem inflammation (25% versus 12%), suggesting a potentially causative role for inflammatory vasculopathy. Third, viral studies employing RNA isolation by qRT-PCR showed both a marked decrease in positive findings, and increase in negative findings (17% and 44%, versus 9% and 22%). Amplification by in situ hybridization (ISH) of signals employing RNAscope™ in tissue sections counterstained by hematoxylin and eosin (H&E) with comparison to control antisense probes failed to show positive SARS-CoV-2 in parenchymal brainstem tissues. Brain microglia activation so noted in 21% of cases was mentioned only rarely in the earlier cohorts.

The findings of a recent cohort of 173 decedents reported by Younger [4] continued the demographic trend of older subjects in a men to women ratio of 2:1, 72% of whom were > 65 years age (range, 0–95 years). Global hypoxic ischemic changes were seen in 42 cases (24%). SARS-CoV-2 virus was isolated in one case among 60 other negative cases. Inflammatory microvasculopathy comprising acute neutrophilic vasculitis (ANV), so noted in 36 consecutive autopsies, was seen in addition to T-cell perivasculitis, with focal or diffuse interstitial and leptomeningeal inflammation in 25 others, totaling 61 cases (35%) overall. Microglial activation and microglial formation was observed in 18 cases (9%).

Taken together, the resulting final cohort of 464 decedents shown in Table 1 affirms five salient findings. First, hypoxia-ischemia in up to one-half of cases overall did not account for all relevant neuropathological features of severe COVID-19. Second, patients presenting with elevated levels of cytokines, increased D-dimer and markers of hypercoagulability are at risk for thrombotic and hemorrhagic parenchymal tissue infarction. Third, unexpected interstitial brainstem inflammation in conjunction with activated microglial nodules suggests the importance of suspected brainstem encephalitis in critically ill patients. Fourth, the discrepancy between PCR and IHC techniques, with absence of SARS-CoV-2 genomes and its proteins with more refined methodologies, suggests the absence of replicating virus in brain tissue with other possible sources of virus including bloodstream contamination across permeable blood–brain or blood-CSF barriers due to systemic inflammation, as well as along leptomeningeal vessels or parenchymal and petechial hemorrhages. Fifth, despite the overall favorable prognosis, systemic and CNS vascular inflammation involving activated innate, humoral and cell-mediated immunity contributes to mortality of COVID-19 neurological illness across all ages. Other factors influencing survival or adverse outcome of COVID-19 infection include comorbidities such as hypertension, cancer, non-SARS-CoV-2 infection, cardiopulmonary failure and other poorly understood or confounding factors.

**Table 1:** Clinical and neuropathologic findings of COVID-19 fatalities

Category	# of Cases, N=464 (%)
Clinical Presentation	
Sex	
Male	256 (68.4%)
Female	118 (31.5%)
Not Reported	93
Age (years)	
<21	12 (5.7%)
21–49	21 (10%)
50–64	54 (25.8%)
>65	122 (58.3%)
Not Reported	258
Serum cytokine and procoagulant levels	
Elevated	31 (91.1)
Normal	3 (9.6%)
Not Reported	433
Duration of hospital illness to death (days)	
0–1	9 (8%)
1–10	47 (41.9%)
>10	56 (50%)
Not Reported	355
Place of death	
Hospital	147 (87.5%)
Nursing Home	11 (6.5%)
Home	10 (6.2%)
Not Reported	299
Cause of death	
Cardiopulmonary failure	155 (83.7%)
Multisystem organ failure	25 (13.5%)
Massive intracranial hemorrhage	3 (1.6%)
Pulmonary embolism	2 (1%)
Not Reported	282
SARS-CoV-2 RNA reactivity in brain sections	
Negative	158 (80.2%)
Positive	39 (19.7%)
Not Reported	240
Histopathology	
Hypoxic ischemia changes and neuronal loss	110 (23.7%)
Acute microscopic ischemic infarcts	66 (14.2%)
Interstitial brainstem inflammation with neuronal loss	53 (11.4%)
Activated microglial nodules	51 (11.0%)
Leptomeningeal inflammation	49 (10.5%)
Focal perivascular parenchymal T-cell infiltrates	40 (8.6%)
Acute neutrophilic endotheliitis	36 (7.7%)
Acute microscopic hemorrhagic infarcts	24 (5.1%)
Petechial hemorrhage	13 (2.8%)
AB deposits	10 (2.1%)
Multisystem inflammatory syndrome	7 (1.5%)
Necrotizing vasculitis	1 (0.2%)
No abnormalities	3 (0.6%)
Associated findings	
Alzheimer disease	16 (0.3%)
Chronic infarction	11 (0.2%)
Lewy body disease	8 (0.1%)
Metastatic cancer	2 (0.004%)
Primary brain tumor	1 (0.002%)
Multiple sclerosis	1 (0.002%)

Other investigators [5–7] have addressed COVID-19 neuropathology in FFPE sections from other vantage points, the results of which broadly support the present insights. Ruz-Caracuel and colleagues [5] studied neuroinflammation in a retrospective single center series of 13 fatal COVID-19 patients of mean age 66 years (52–83 years) and mean length of stay of 34 days (3–102 days) noting widespread neuroinflammation and activated microglia in all cases that did not correlate with length of ICU or hospital stay, IL-6 levels or a clinical parameter of systemic macrophage activation; nor was there evidence of positive staining for SARS-CoV-2 by qRT-PCR. Humayun and coworkers [6], who conducted a case–control study of acute brain injury (ABI) and neuroinflammation, found a higher frequency of vasculopathy in COVID-19 (10 cases) compared to non-COVID-19 ARDS (20 cases) (60% versus 25%) with a trend toward increased fatal events of infratentorial vascular pathology including ischemic infarcts, edema and herniation. Magaki and coinvestigators [7] compared the literature of the human immune deficiency virus (HIV) and COVID-19 pandemics, noting absence of SARS-CoV-2 RNA or proteins in brain tissue by RNAscope and IHC or cultured in cerebrospinal fluid, compared to the presence of HIV-1 antigens in CNS tissue, macrophages, microglia and multinucleated giant cells by IHC and ISH. Unlike HIV, it was unclear whether SARS-CoV-2 was neurotropic or neuroinvasive, although mechanisms other than direct infection likely contribute to both acute and chronic COVID-19 illness.

Looking forward, the importance of performing autopsy studies in COVID-19 decedents resides not only in tabulating disease pathology and understanding key mechanisms of disease, but in conceptualizing complementary experimental studies to facilitate critical knowledge that may improve daily clinical practice [8]. Precise methodology involving RNAScope suggests that post-infectious immunity, and not in situ viral replication, accounts for morbid neurological illness and predominates at the time of death. There is a proposal for the use of IHC using antibodies against SARS-CoV-2 nucleocapsid to yield the highest sensitivity and specificity (0.72 and 0.95 respectively), showing a linear relationship between the presence of viral proteins by IHC and RT-qPCR SARS-CoV-2 viral RNA load (Pearson correlation coefficient r = −0.83, *p*-value < 0.0001, based on *n* = 35) [9]. The pathogenic origin of acute neutrophilic vasculitis encountered in 36 (7%) of decedents is not well understood, but may reflect the selective vulnerability of the microcirculation early in the illness, associated with hypoxia, circulating cytokines, and renin-angiotensin system dysfunction.

There are several obvious study limitations. First, this was not a scientifically or neuropathologically rigorous study due to missing information, variable case sizes, different autopsy laboratories, and different time periods between 2020 and 2024. Second, it was not possible to attribute any particular abnormal pathological finding to COVID-19-related illness in the absence of control tissue. Third, the summary data of Table 1 provides a snapshot of the cumulative features of morbid COVID-19 over the span of the pandemic, taken out of context of the timing of vaccination and across all available treatments. Fourth, the meta-analysis was conducted by a single author with biases in data collection and in addressing particular research questions.
